# Computed tomography angiography of liver alveococcosis: a case report

**DOI:** 10.1186/s13256-024-04399-1

**Published:** 2024-03-11

**Authors:** Saodatkhon Magzumova, Umidjon Isroilov, Nigora Djuraeva, Zarina Khaybullina, Aybek Amirkhamzaev, Nargiza Vakhidova, Khanum Abdukhalimova, Alisher Sultanov, Bekhzod Abdullaev

**Affiliations:** 1 Department of Radiology, National Specialized Scientific and Practical Medical Center for Surgery Named After Academician V.Vakhidov, Kichik Khalqa Yuli 10/1, 100115 Tashkent, Uzbekistan; 2 Department of Biochemistry and Microbiology, National Specialized Scientific and Practical Medical Center for Surgery Named After Academician V.Vakhidov, Kichik Khalqa Yuli 10/1, 100115 Tashkent, Uzbekistan; 3 Research Department of Biotechnology, New Uzbekistan University, Movarounnahr 1, 100007 Tashkent, Uzbekistan

**Keywords:** Liver alveococcosis, Contrast enhanced computed tomography, Liver lesions, Cystic formation

## Abstract

**Background:**

Alveococcosis, helminthiasis caused by the larvae of *Alveococcus multilocularis*, is characterized by the formation of parasitic nodes in the liver. This clinical case is a rare occurrence of liver alveococcosis in Uzbekistan.

**Case presentation:**

We present a case of a 33-year-old Asian woman from Uzbekistan who complained of discomfort in the epigastric region and right hypochondrium, along with general weakness. She had been experiencing symptoms for 5 months when a routine ultrasound examination revealed a structural formation in the liver. Patient was investigated by using contrast enhanced computed tomography (CT) and diagnosed with liver alveococcosis with multiple lesions. Patient underwent diagnostic laparotomy with obtaining gross specimen, biomaterial was examined microscopically and found that there were small fragments of fibrous tissue determined together with small groups of cystic formations and walls consisted of chitin. Moreover, contrast enhanced CT allowed us to differentiate liver alveococcosis from cavernous hemangioma, hepatocellular carcinoma, and liver metastases from unknown source.

**Conclusion:**

Contrast enhanced CT plays a major role in differentially diagnosing liver alveococcosis and makes it the first line method of choice for the consideration of the future treatment and surgical interventions.

## Introduction

Liver alveococcosis (LA) is a rare but potentially life-threatening serious parasitic disease. It develops because of intrahepatic growth of Echinococcus multilocularis larvae. This parasite induces liver lesions, and it is somewhat may vary from echinococcosis caused by Echinococcus granulosus, a hydatid species that is common worldwide [1]. According to the World Health Organization (WHO), alveococcosis is one of the most frequent infection in tropical zone of Northern Hemisphere of the world. In the past 100 years it is increasingly integrated to the global economy and has accentuated a wide distribution [2]. However, it is considered as one of the most dangerous and very rare parasitic disease with high mortality and morbidity in Uzbekistan. LA diagnosis depends on clinical findings, medical history, radiologic imaging modalities, laboratory examinations, and histopathology verification. At least two criteria should be present for diagnosing LA. First, in imaging should be shown characteristic lesions. Secondly, specific serum antibodies should be detected against Echinococcus antigens. Thirdly, pathologic verification of E. multilocularis metacestodes and last criteria is existence of parasite nucleic acids in clinical specimens [3].

WHO classification of alveolar echinococcosis was developed as the PNM system by WHO Informal Working Group on Echinococcosis (WHO-IWGE), which is mainly based on imaging findings (Table 1) [4].

**Table 1:** PNM system for classification of human alveolar echinococcosis [3]

P	Hepatic localization of the primary lesion	
	PX	Primary lesion cannot be assessed
	P0	No detectable liver lesion
	P1	Peripheral lesions without proximal vascular and/or biliary involvement
	P2	Central lesions with proximal vascular and/or biliary involvement of one lobe^a^
	P3	Central lesions with hilar vascular and biliary involvement of both lobes and/or with involvement of two hepatic veins
	P4	Any lesion with extension along the portal vein, inferior vena cava, or hepatic arteries and the biliary tree
N	Extra hepatic involvement of neighboring organs or tissues^b^	
	NX	Cannot be evaluated
	N0	No regional involvement
	N1	Regional involvement of contiguous organs or tissues
M	Absence or presence of distant metastases^c^	
	MX	Not completely evaluated
	M0	No metastasis
	M1	Metastasis present

^a^For PNM classification, the plane projecting between the bed of the gallbladder and the inferior vena cava divides the liver in two lobes

^b^Neighboring organs and tissues include the diaphragm, lungs, pleura, pericardium, heart, gastric and duodenal wall, adrenal glands, peritoneum, retroperitoneum, parietal wall (muscles, skin, bone), pancreas, regional lymph nodes, hepatic ligaments, and kidney

^c^Distant metastasis locations include the lungs, distant lymph nodes, spleen, kidney, central nervous system, orbits, bone, skin, muscle, distant peritoneum, and retroperitoneum

LA presents with a wide variety of CT-morphological manifestations [5]. CT is the primary imaging modality for detecting anatomic and morphologic features of lesions and predicts the characteristic signs of calcification, as well as determining typical calcifications. It is one of the most effective methods to evaluate liver damage and vascular/bile duct structures. It also helps investigate the number, size, and location of organ damage and allows a comprehensive preoperative evaluation of vascular, biliary, and extrahepatic extension, which is significant consideration when evaluating damage resectability [6]. The presence of multiple LA is associated with only one primary morphological pattern. In rare cases, multiple lesions in liver may be characterized by more than one primary morphology and these must be evaluated separately over the course of the disease [5].

In this case, the nodes can be from half a centimeter to 30 cm in diameter. In addition, there were cases of extrahepatic metastasis, when the alveococcal bladder reached the edge of the liver and grew into adjacent adjacent organs, including the diaphragm, kidney, bone and other tissues—exophytic growth. Therefore, LA can be justifiably called “parasitic cancer”. If a breakthrough occurs, then the contents enter the abdominal, pleural cavity or hollow organs. We present the case of a 33-year-old woman from Uzbekistan, where LA is non-endemic region, who was diagnosed with liver alveococcosis. To our knowledge, this is the first case report of LA diagnosed by CT angiography from Uzbekistan.

## Case presentation

### Anamnesis vitae

A 33-year-old Asian woman from Uzbekistan presented with complaints of epigastric and right hypochondrium discomfort, along with general weakness, upon admission. According to the patient, she had been experiencing these symptoms for the past 5 months, and the discovery of a liver structural abnormality during a routine ultrasound examination led to her referral to the Republican Surgery Center. The patient reported no history of hepatitis, use of hormonal preparations, past injuries or surgeries, or any other known medical conditions. There were no reported allergies to medications or food products. The complete blood count showed no pathological changes. Smoking, alcohol or drug abuses have not been determined. There were not detected any non-communicable diseases, patient has not reported any additional diseases or complaints, no hormone replacement therapy.

### Family history

Her father lives with hypertension, has had coronary artery disease. Her husband has no any specific disease or complaints. She has 2 sons with normal health status.

### Social history

Patient graduated high school and has not continued education, currently she does not work. Patient feels safe and well-cared for in his home. Patient denies any recreational drug use. Patient is sexually active and reports a monogamous relationship.

### Organ based anamnesis

Skin was negative for photosensitivity, easy bruising, skin discoloration, new or changing moles, ulcers, hair loss, dry or brittle nails, or dry skin. Head and face were without traumatic injury, ptosis, and loss of sensation. Respiratory, sensory, neural, cardiovascular, urogenital, psychological and endocrine systems were examined and was not found any significant symptom.

### Clinical symptoms and lab examinations

Patient has normal temperature, heart rate was 68 per minute, and arterial blood pressure was 110/70 mm Hg, respiratory rate was16 per minute. The clinical symptoms changed during the stay in hospital. Jaundice and epigastric pain are the primary symptoms in hepatic invasion, but weight loss and malaise also developed later. Vascular structure and bile ducts invasion have been reported. Routine laboratory tests do not reveal any specific results. Erythrocyte sedimentation rate was increased, eosinophilia, hypergammaglobulinemia was also detected in laboratory findings. Immunodiagnostic tests have been done for confirming diagnosis of LA due to presence of more specific antigens. The best option for determination of serum antibodies in LA case was to use purified antigens such as alkaline phosphatase (AP) C-125 antigen.

Biochemical analysis revealed moderate deviations from normal ranges. Gamma-glutamyl transferase level was 66 U/L (reference range for adults is from 5 to 40); alkaline phosphatase level was 203 IU/L (normal range between 44 and 147 IU/L). The CA-125 was moderately elevated at 38.9 U/mL (normal range between 0 and 35 U/mL). A puncture biopsy of the liver indicated histological findings of necrotic tissue and blood (Table 2).

**Table 2:** Laboratory findings of the patient with alveolar echinococcosis

Indicators	Results	Normal range
ALT (IU/L)	115	19–25
AST (IU/L)	99	8–33
T & D Bilirubin (mg/dL)	45/28	0.1–1.2 (T) > 0.3 (D)
PT (sec)	17.4	11–13.5
Albumin (g/dL)	2.6	3.4–5.4
Alkaline phosphatase (IU/L)	203	44–147
Gamma-glutamyl transferase (U/L)	66	5–40
CA-125 (U/mL)	38.9	0–35
Total cholesterol	159	125–200
Low-density lipoprotein cholesterol (mg/dL)	78	50–100
High-density lipoprotein cholesterol (mg/dL)	65	> 60

*ALT* Alanine transaminase, *AST* Aspartate transaminase, *T&D Bilirubin* Total and Direct Bilirubin, *PT* Prothrombin time Albumin, *CA-125* Cancer Antigen 125, *IU/L* international units per liter, *Mg/dl* milligrams per deciliter, *g/dl* grams per deciliter, *U/L* units per liter, *U/ml* units per milliliter

### Diagnostic imaging

Ultrasound examination revealed the following measurements: the right lobe of the liver measured 13.6 cm, while the left lobe measured 8.4 cm. The liver exhibited an echo-homogeneous structure with seals along the bile ducts, which were not dilated. Structural formations with uneven contours and heterogeneous contents were visualized in the V–VIII segments, measuring 11.5 × 8.3 cm and exhibiting increased echogenicity. An anechoic formation measuring 7.8 × 6.7 cm was observed in the VI segment of the liver.

**Fig. 1:**
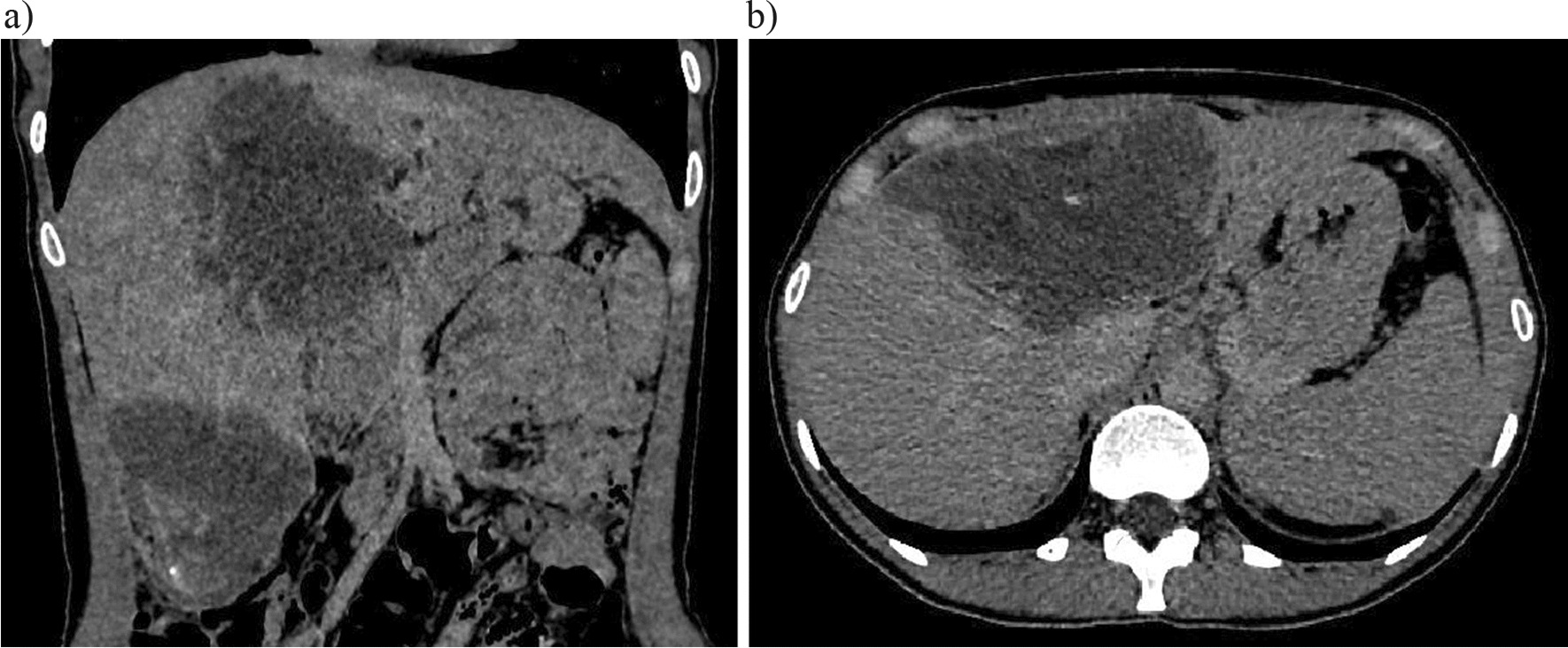
Coronal (**a**) and axial (**b**) CT image in the native phase demonstrates tumor masses in IV, VIII and partially V, VI segments. Uneven fuzzy contours, signs of infiltrative growth into the liver parenchyma, cystic-solid structure with + 10 to + 40 HU, single calcified inclusions (+ 145HU), invasion into the Glisson’s capsule, dilatation of the bile ducts along the tumor periphery

**Fig. 2:**
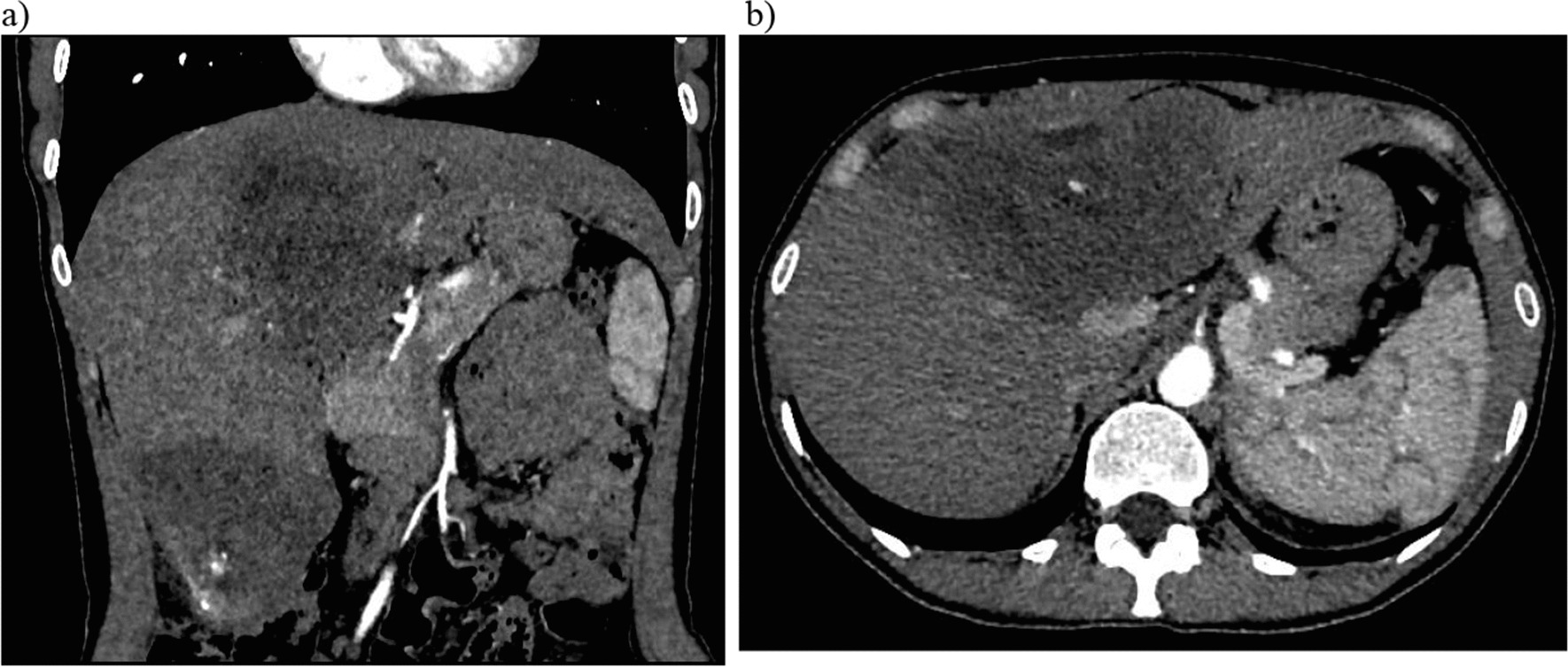
Contrast-enhanced coronal (**a**) and axial (**b**) CT images show in arterial phase there is a moderate accumulation of contrast agent in the solid part density up to + 45 to + 55 HU

**Fig. 3:**
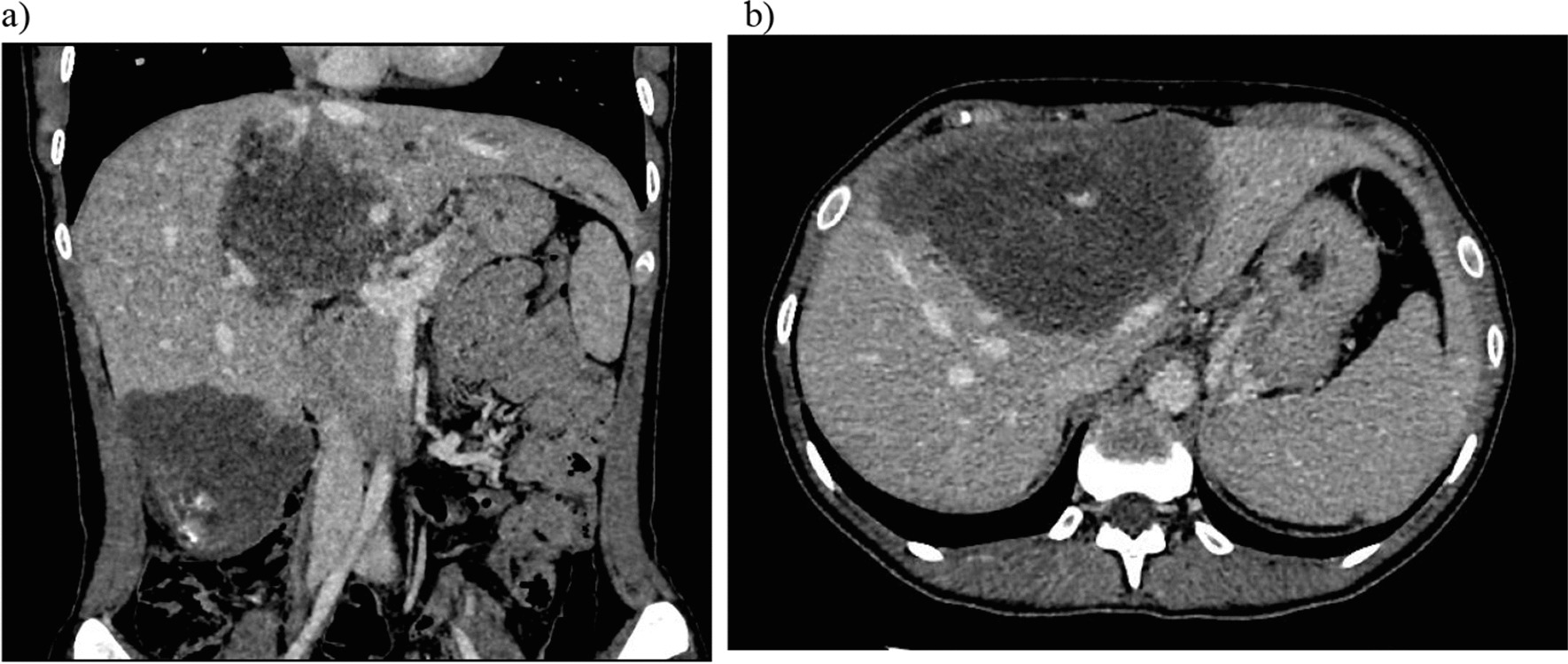
**a**, **b**. In the venous phase: continued moderate accumulation of contrast agent in the solid part of the formation density up to + 45 to + 65 HU

**Fig. 4:**
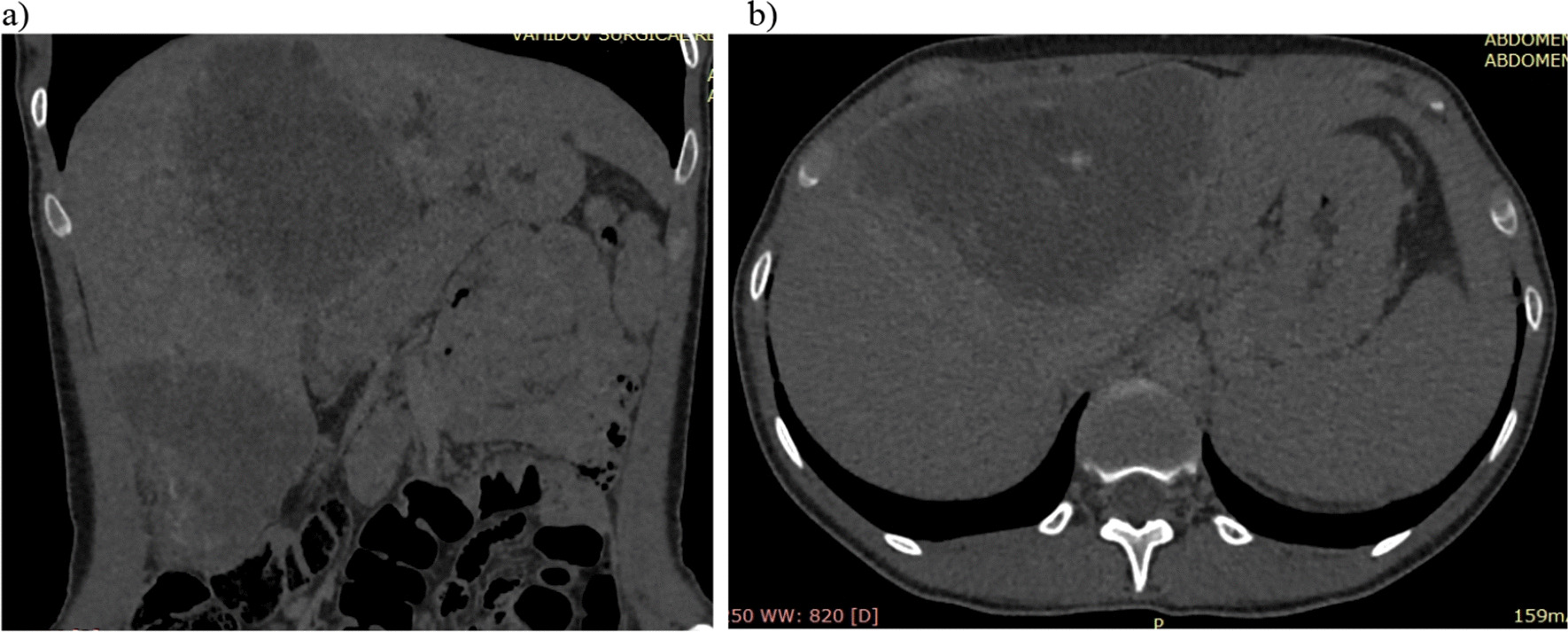
**a**, **b**. Delayed phase—preservation of CV in the solid part of the formation—density up to + 40 to + 60 HU

Patient underwent contrast enhanced CT (Figs. 1, 2, 3, 4). In IV, VIII and partially V segments of the liver was detected irregularly shaped volumetric formation with signs of infiltrative growth in the surrounding parenchyma of the liver, with fuzzy uneven contours, cystic-solid structure, heterogeneous density from + 10 to + 40 HU. Calcification areas and small cysts were determined in the structure of lesion; the size was up to 12–15 mm in diameter, with moderate bile duct ectasia along the periphery. Glisson’s capsule can be traced intermittently at this level. A similar mass measuring 8 × 7 × 7 cm is determined in the VI segment, connection with the upper pole of the right kidney, loops of the hepatic flexure of ascending colon, parietal peritoneum.

### Diagnostic surgery technique

Patient undervent diagnostic laparotomy under the general anesthesia. Diagnostic laparotomy was performed by Fyodorov approach which incision was made 15 cm from the right upper quadrant. The optimal incisions are in the right hypochondrium, providing wide access to the liver segments, gallbladder, extrahepatic bile ducts and duodenum. An oblique incision of the abdominal wall is made 2 cm below and parallel to the right costal arch (according to Fyodorov). The hepatoduodenal ligament is divided into the cystic, hepatic and common bile ducts. During the investigation it was determined that lesion spreads in the projection of liver segments 4a-4b to the S8-S3 segments, as well as detected a large formation of dense-grey consistency of a purple color, with a focus of a light yellow surface, and a compaction in the center, measuring 13x15 cm. A similar formation was located in the S5 projection with a transition to S6. In abdominal cavity, there was no organic pathology. It was considered that lesion is unresectable and sufficient volume of residual parenchyma was not exist.

### Treatment and follow-up

Patient admitted to the hospital with the diagnosis of liver alveococcosis P4N0M0, uncomplicated stage. Length of stay at the hospital was 11 days. Patient received antiparasitic therapy: Albendazole 400 mg twice a day. Surgical intervention was not recommended because it could lead to the hepatic failure and bleeding. After discharge from hospital patient did not come back or approach to the hospital. All acts for contacting her were without any results. That’s why the authors cannot give any feedback about long term follow-up after the hospital discharge.

## Discussion

Alveococcosis is very rare disease which is diagnosed in Uzbek population and human incidence rates for alveococcosis is not clear. This case is the first one which was officially registered in Uzbekistan and reported to the public. Before that, alveococcosis was not registered and published anywhere in Uzbekistan. The uniqueness of this case is that multiple lesions were in two lobes of liver and it could be treated by only liver transplantation. Because of huge mass of parasitic nodules resection or radical operation was inefficient.

Alveococcosis is a helminthiasis belonging to the tenidosis group, caused by larvae of *Alveococcus multilocularis*. It is characterized by the development of parasitic nodules within the liver. These nodules exhibit tumor-like masses with indistinct borders and heterogeneous content, consisting of scattered hyperdense areas of calcification and hypodense areas corresponding to regions of necrosis [7]. The calcifications were often referred as “lime splashes” and display high density (+ 420 HU). Primary extrahepatic locations of alveococcus are exceedingly rare. Density of granulation tissue and fibrotic area of lesion increases, most accordingly at the venous portal phases, but these areas still remain hypodense hepatic lesions in comparison with the intact liver parenchyma. Small cavities and foci of necrosis with liquefaction do not change their densitometric parameters and are more clearly visualized on post-contrast scans [8, 9].

Diagnosing liver alveococcosis is significant by differentiating from cavernosis hemangioma, hepatocellular carcinoma, and metastatic cancer with unknown primary site [10]. Echinococcosis manifests as a cystic structure encompassed by a multilayered capsule, often with multiple chambers. The capsule may exhibit varying degrees of calcification, and septate structures are commonly observed along with endogenous daughter cysts [11]. Echinococcosis typically initiates a primary lesion in the lungs [12]. Cavernous hemangioma, another differential diagnosis, presents as a hypodense formation within the liver parenchyma during native studies [13]. In the arterial phase, there is nodular accumulation with contrast enhancement at the periphery of the lesion. During the venous phase, there is progressive centripetal accumulation of contrast within the lesion [14]. Hepatocellular carcinoma (HCC), characterized by multinodular mass formations, demonstrates pronounced contrast enhancement in the late arterial phase. However, rapid contrast washout during the portal phase results in the tumor appearing isodense or hypodense relative to the surrounding healthy liver parenchyma [8].

The primary techniques for diagnosing LA are radiological imaging techniques. According to the literature Ultrasonography methods are the best choice for diagnosis and screening of LA. However, the results are highly dependent on the skill and knowledge of the ultrasound operators. In this case CTA was useful to determine the characteristics of lesions, invasion of the contiguous structures and the presence of secondary extra-hepatic metastases in the organism.

The preferred treatment method for AE is resection of affected parenchyma of the liver. It is not advisable to have conservative or palliative surgery as there is no benefit compared to pharmacotherapy. Albendazole is the most effective drug for patient care in the preoperative period and also for the patients with contraindications to surgery and. Using Albendazole increases the long term survival for patients.

### Learning points

1. Contrast enhanced CT has significant advantages in diagnostics of liver alveococcosis, making it the first line method of choice in order to diagnose and necessary before surgery.
2. The latent stage of liver alveococcosis can persist for years before symptoms appear; in this patient the disease was incidentally detected.
3. Contrast enhanced CT allows us to illustrate the distinctive pattern of calcification and enables anatomic and morphologic characterization of lesions. Additionally, it helps to determine size, location and number of the lesions. It is possible to determine vascular, biliary, and extrahepatic extension in a preoperative assessment, which is essential to consider when evaluating resectability of the lesion.

## Conclusion

This clinical case is a rare occurrence of liver alveococcosis in Uzbekistan. In non-contrast enhanced CT, liver alveococcosis demonstrates that liver lesions come with signs of indistinct boundaries, heterogeneous cystic-solid structure, single calcified inclusions, infiltrative growth of the lesion. However, it is very difficult to differentiate from other liver diseases like cancer and metastasis. For the differential diagnosis the most important diagnostic method is contrast enhanced CT and it allows differentiating liver alveococcosis from cavernous hemangioma, hepatocellular carcinoma and metastases from unknown sources.

**Fig. 5:**
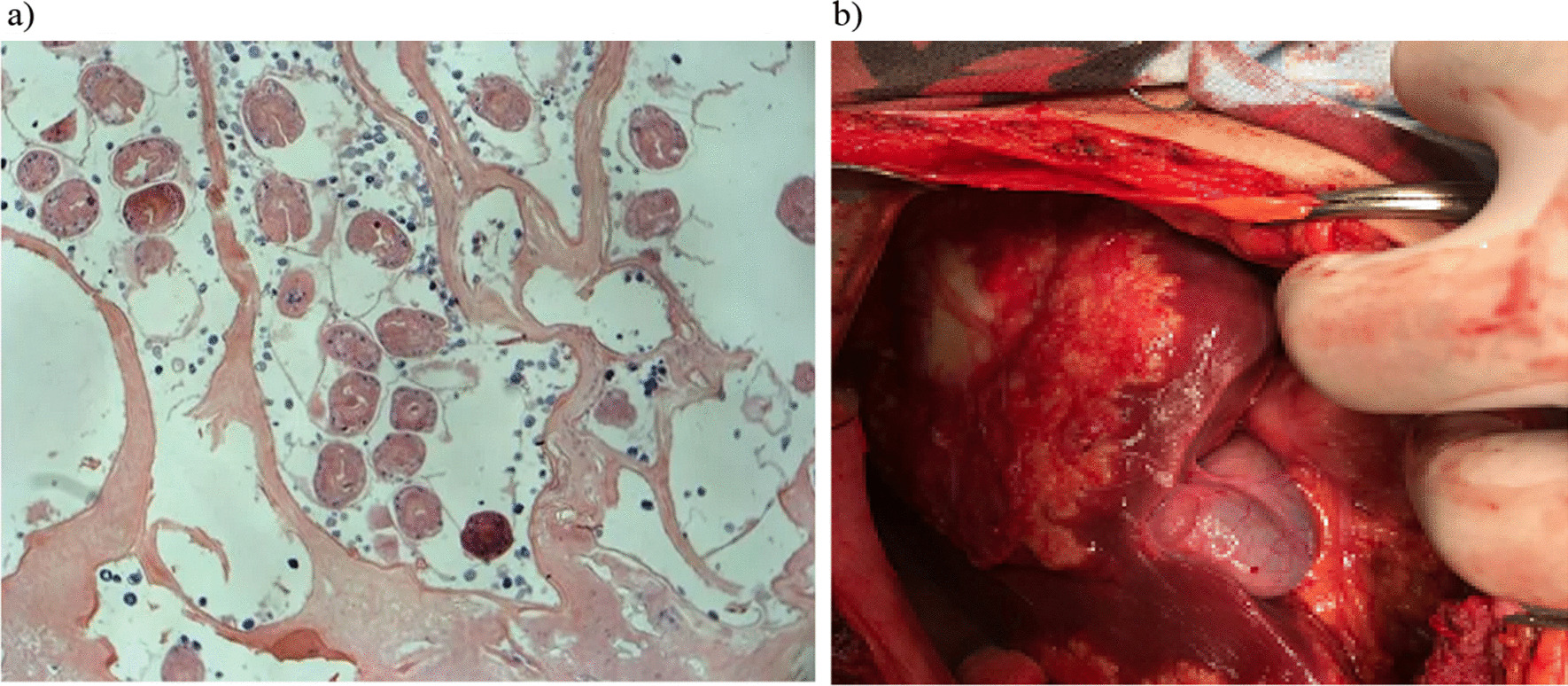
**a**, **b** after diagnostic laparotomy, histologic examination was carried out. Diagnostic laparotomy was done on February 13, 2019. Histologic investigation of the gross specimen showed that small fragments of fibrous tissue was determined together with small groups of cystic formations and walls consisted of chitin. Diagnosis: Chitinous membrane of alveococcocus

Moreover, absence of the neoplasm on contrast enhanced CT also confirms this diagnosis (Fig. 5). Thus, if a parasitic etiology of liver mass is suspected, contrast enhanced CT of the liver can be considered the most appropriate method for differential diagnosing and must be the mandatory method of investigation of these types of patients.

## Data Availability

Not applicable.
